# Quantitative Modeling of the Degradation of Pesticide Residues in Wheat Flour Supply Chain

**DOI:** 10.3390/foods12040788

**Published:** 2023-02-13

**Authors:** Zhiqian Ding, Meirou Lin, Xuelin Song, Hua Wu, Junsong Xiao

**Affiliations:** 1 College of Food and Health, Beijing Technology & Business University (BTBU), Beijing 100048, China; 2 COFCO Grains Holding Co., Ltd., Beijing 100020, China; 3 College of Chemistry and Materials Engineering, Beijing Technology & Business University (BTBU), Beijing 100048, China

**Keywords:** wheat and flour, pesticide residues, storage conditions, ultra-high performance liquid chromatography–tandem mass spectrometry (UPLC-MS/MS), QuEChERS

## Abstract

Pesticide residues in grain products are a major issue due to their comprehensive and long-term impact on human health, and quantitative modeling on the degradation of pesticide residues facilitate the prediction of pesticide residue level with time during storage. Herein, we tried to study the effect of temperature and relative humidity on the degradation profiles of five pesticides (carbendazim, bensulfuron methyl, triazophos, chlorpyrifos, and carbosulfan) in wheat and flour and establish quantitative models for prediction purpose. Positive samples were prepared by spraying the corresponding pesticide standards of certain concentrations. Then, these positive samples were stored at different combinations of temperatures (20 °C, 30 °C, 40 °C, 50 °C) and relative humidity (50%, 60%, 70%, 80%). Samples were collected at specific time points, ground, and the pesticide residues were extracted and purified by using QuEChERS method, and then quantified by using UPLC-MS/MS. Quantitative model of pesticide residues was constructed using Minitab 17 software. Results showed that high temperature and high relative humidity accelerate the degradation of the five pesticide residues, and their degradation profiles and half-lives over temperature and relative humidity varied among pesticides. The quantitative model for pesticide degradation in the whole process from wheat to flour was constructed, with R^2^ above 0.817 for wheat and 0.796 for flour, respectively. The quantitative model allows the prediction of the pesticide residual level in the process from wheat to flour.

## 1. Introduction

Wheat flour is one of the most common food ingredients in the world, and it occupies an important position in the food industry [1,2]. Pesticides are commonly used in wheat production and play a positive role in reducing crop yield losses caused by crop diseases, pests, and weeds [3]. The general population is exposed to pesticides primarily through eating food contaminated with pesticides residues. Although these pesticides are developed to function with minimal impact on human health, long-term exposure to pesticide residues in food remains a major risk. Controlling pesticide residues in the supply chains of food, especially rice and wheat flour, is important, and the ability to predict their degradation under various environmental conditions and processing factors is vital to their control [4]. Thus, in this research, the degradation profiles of commonly used pesticides in the supply chain of wheat flour, mainly including the storage and milling period of wheat flour, were studied.

The common pesticides in wheat include fungicides (e.g., carbendazim, chlorothanlonil, carboxin, cyproconazole, and difenoconazole), herbicides (e.g., bensulfuron methyl, carfentrazone-ethyl, dicamba, flupyrsulfuron methyl, and difenoconazole), insecticides (e.g., chlorpyrifos, triazophos, carbosulfan, deltamethrin, and esfenvalerate), plant growth regulators (e.g., ethephon, trinexapac, and chlormequat), of which five pesticides (carbendazim, bensulfuron methyl, triazophos, chlorpyrifos, and carbosulfan) are used more frequently in wheat cultivation. Pesticide residue problems often occur in wheat and wheat products [5,6,7].

The commonly used methods for pesticide extraction and purification include dispersive liquid–liquid microextraction, solid-phase extraction, solid-phase microextraction, QuEChERS (Quick, Easy, Cheap, Effective, Rugged, and Safe) method [8,9,10,11]. Among them, the QuEChERS method is one of the most successful methods [12], which is based on the principle of using absorbent filler to interact with impurities in the matrix to adsorb impurities and achieve the purpose of impurity removal. This method is fast, simple, economical, efficient, durable, and safe, which has been widely used in recent years for the study of pesticide residues in food [13].

The commonly used methods for the detection of pesticides in food are gas chromatography, gas chromatography–tandem mass spectrometry, liquid chromatography, liquid chromatography–tandem mass spectrometry, and ultra-high performance liquid chromatography–tandem mass spectrometry (UPLC-MS/MS) [14,15,16,17]. Gas chromatography is incapable of analyzing pesticides with high polarity and poor thermal stability [18]. Liquid chromatography–mass spectrometry is capable of detecting various pesticide residues, but its detection limit is often difficult to meet the requirements of pesticide residue detection [19]. Tandem mass technique, especially triple quadrupole mass spectrometry, coupled with ultra-high-performance liquid chromatography, is more comprehensive, sensitive, stable, and of wide detection range for the quantification of pesticide residues [20].

In this study, the degradation patterns of five typical pesticides during the storage of wheat and flour under various storage conditions were investigated, using QuEChERS method coupled with UPLC-MS/MS. The five pesticides included carbendazim, a broad-spectrum fungicide, bensulfuron methyl, an herbicide, triazophos, an organophosphorus acaricide, chlorpyrifos, a thiophosphate insecticide, and carbosulfan, a carbamate insecticide. A typical wheat supply chain includes the storage of wheat grain, the milling process, and the storage of wheat flour. The degradation profiles of these five pesticide residues, combined with their processing factors during wheat milling, will facilitate the model construction to predict the pesticide residue levels in the wheat supply chain.

## 2. Materials and Methods

### 2.1. Materials and Reagents

**Figure 1:**
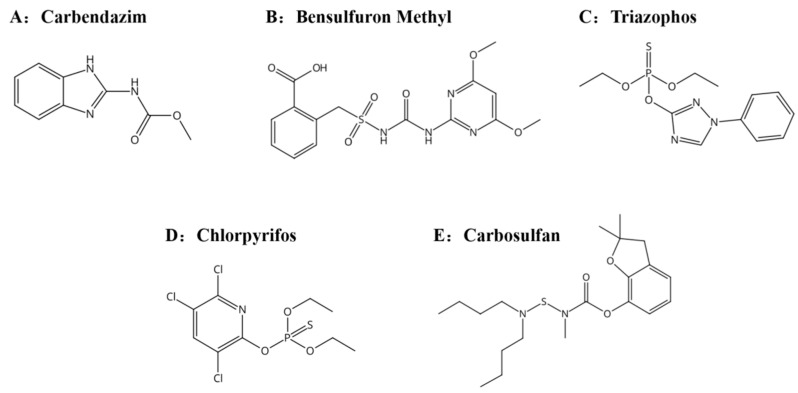
Chemical structures of five pesticides.

Wheat (Jimai 22) was provided by Crop Research Institute, Shandong Academy of Agricultural Sciences (Jinan, China). Flour (Fuqiang) was supplied by Beijing Guchuan Food Co., Ltd. (Beijing, China). The pesticide standards, including triphenyl phosphate (TPP), carbendazim, bensulfuron methyl, triazophos, chlorpyrifos, and carbosulfan (all 99% purity), were purchased from Accustandard Inc. (New Haven, CT, USA). The five pesticides (carbendazim, bensulfuron methyl, triazophos, chlorpyrifos, carbosulfan) chemical structures are shown in Figure 1. Chromatographic-grade methanol and formic acid were purchased from Mreda (Beijing, China), and 0.22 µm nylon membrane filter was purchased from Tianjin jinteng Experimental Equipment Co., Ltd. (Tianjin, China). Waters BEH C_18_ column (100 mm × 2.1 mm, 1.7 μm) was purchased from Waters (Milford, CT, USA). QuEChERS purifier and salt package were purchased from Beijing Dima Outai Science Technology Co., Ltd. (Beijing, China).

### 2.2. Standard Solutions

Stock solutions of each pesticide standard (100 mg/L) in the same volumetric flask were prepared with chromatographic grade methanol. The stock solutions were prepared with methanol as a mixed stock solution (2 mg/L) and stored at 4 °C in a refrigerator for use. A series of concentrations of working solutions (0.005, 0.020, 0.050, 0.200, 0.400, 1.000 mg/L) were obtained by sequentially diluting the mixed stock solution with wheat and flour blank matrix solution.

### 2.3. Preparation of Positive Samples

According to the standard “Maximum Residue Limits of Pesticides in Food of National Food Safety Standard” (GB 2763-2021), and the limit of detection (LOD) and limit of quantification (LOQ) of the experiment, a mixed solution containing 10-fold maximum residue limit (MRL) of carbendazim, triazophos, and carbosulfan, 1-fold MRL of chlorpyrifos, and 20-fold MRL of bensulfuron methyl were prepared in methanol. Then, 5.0 g of each wheat and flour samples were sprayed with the above prepared mixed solution and left sealed for 24 h for storage experiments.

### 2.4. Control of Sample Storage Conditions

Different concentrations of glycerol were prepared and placed into closed drying containers to obtain environments with relative humidity of 50%, 60%, 70%, 80%, respectively. The wheat and flour samples sprayed with pesticides were placed in containers with different humidity control, and then each container was placed in incubators with constant temperature at 20 °C, 30 °C, 40 °C, and 50 °C, respectively. Different combinations of temperature and humidity were obtained.

### 2.5. Extraction and Purification of Samples

The extraction and purification methods for wheat and flour were based on the classical QuEChERS method with minor change [21,22,23]. Wheat and flour samples were ground and weighed (5 ± 0.02 g) in a polypropylene centrifuge tube (50 mL), and TPP (100 μL) and pure water (10 mL) were added to mix, and the samples were left for 10 min to infiltrate. After immersion, samples were extracted by adding 10 mL methanol and salt packets (1.5 g CH_3_COONa, 6 g anhydrous MgSO_4_) and vortexed to mix [24,25,26]. After centrifugation at 4000× *g* for 10 min, the supernatant (6 mL) was collected in a centrifuge tube (10 mL). The samples were purified by adding a purifier (400 mg PSA, 400 mg C18, anhydrous MgSO_4_) to the supernatant, and then vortexed and mixed. After centrifugation at 2000× *g* for 10 min, the supernatant (2 mL) was aspirated into a new centrifuge tube (10 mL). After blowing nitrogen to near-dryness at 40 °C, 2 mL methanol was added to redissolve, and then vortexed and mixed. Finally, the purified extracts were filtered through a 0.22 µm nylon membrane filter to analyze with UPLC-MS/MS.

### 2.6. Conditions for the UPLC-MS/MS Analysis

**Figure 2:**
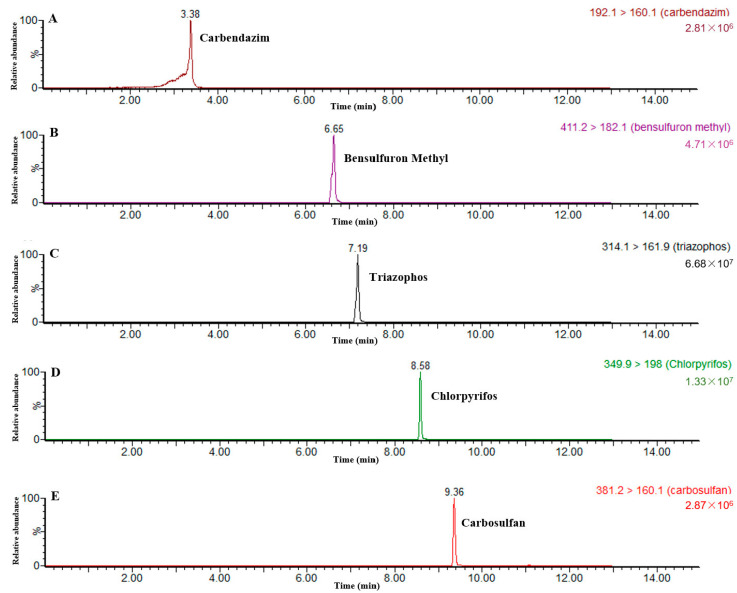
Chromatograms of quantitative ion pairs of five pesticides. Top to bottom: (**A**) Carbendazim, (**B**) Bensulfuron methyl, (**C**) Triazophos, (**D**) Chlorpyrifos, and (**E**) Carbosulfan.

The five pesticides were separated on a UPLC-MS/MS (Waters ACQUITY UPLC I-Class/Xevo TQ-S) (Waters, Milford, CT, USA) equipped with positive mode (ESI+) and Waters BEH C_18_ column (100 mm × 2.1 mm, 1.7 μm). The mobile phase consisted of 0.1% formic acid in a mixed solvent of water (A) and acetonitrile (B). The gradient elution procedure was as follows: 10% B (0–1.5 min), 50% B (1.5–4 min), 90% B (4–10.5 min), and 10% B (10.5–13 min). The flow rate of the mobile phase was set at 0.4 mL/min, and the injection volume was 5 µL. The samples were measured by multiple reaction monitoring (MRM) in positive ion. The MRM parameters are shown in Table 1. The parameters of MS detection were as follows: capillary voltage, 3.0 KV; ion source temperature, 150 °C; desolvation temperature, 500 °C; desolvation air flow, 800 L/h; and cone voltage, 35 V. The quantitative ion chromatograms for the five pesticides are shown in Figure 2.

### 2.7. Data Analysis

The data of this study were collected under Masslynx 4.1 software (Waters Corp., Milford, MA, USA). Microsoft Excel 2020 software (Microsoft Corp., Redmond, WA, USA) was used for preliminary sorting of experimental data, and Minitab 17 software (Minitab Inc., State College, PA, USA) was used for response surface analysis. Origin 2019b software (OriginLab Corp., Northampton, MA, USA) was used to draw plots and calculate the Area Under Curve (AUC).

## 3. Results and Discussion

### 3.1. Standard Curves and Methodological Validation

In this study, the standard curves and coefficients of determination (R^2^) of the five pesticides in wheat and flour blank matrices were obtained using the internal standard method. As shown in Table 2 and Table 3, the R^2^ of the standard curves of the five pesticides were all greater than 0.9990, indicating good linearity in a certain linear range.

The LOD was obtained according to a three-fold Signal-to-Noise (S/N) ratio, and the LOQ was obtained according to a ten-fold S/N ratio [27]. Spike recoveries of the five pesticides were obtained by spiking the samples, wheat or flour, with a mixed standard solution of five pesticide residues at the level of 0.05 mg/kg. The experiment was set up in five parallel, and the results are shown in Table 4. The LOD and LOQ of five pesticide residues were 0.001~0.005 mg/kg and 0.002~0.01 mg/kg, respectively. The matrix spike recovery rate of pesticide residues was 86.77%~106.28%, and the precision was 2.88%~6.76%. It showed that the method has good recovery and precision and meets the requirements of pesticide residue quantification. This method can be used for the following experiments.

### 3.2. Critical Points of Pesticide Residue Change in Wheat Flour Supply Chain

The supply chain from wheat to flour includes raw material storage, cleaning, milling, sieving, cleaning, final product storage, packaging, and circulation, etc. Through previous literature research and investigation, we found that raw material storage, milling, and final product storage are the critical points in wheat flour supply chain [28,29]. The critical points and their main mechanisms and impact factors pesticide residues are shown in Table 5.

**Figure 3:**
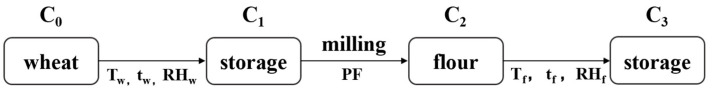
Critical processes in wheat flour processing.

Figure 3 showed the supply chain of wheat flour, from the storage of wheat grain, the milling process of wheat, to the storage of flour. Among them, C_0_, C_1_, C_2_, and C_3_ were pesticide residue concentrations at corresponding stage, and T_w_, t_w_, and RH_w_ are the temperature, time, and relative humidity during wheat storage, and T_f_, t_f_, and RH_f_ are the temperature, time, and relative humidity during storage. The processing factor (PF) was used to describe the effect of milling process on the degradation of pesticides degradation and defined as the ratio of C_2_ to C_1_. PF less than 1 indicates that the processing method can effectively reduce the amount of pesticide residues, and the lower the PF value, the lower the amount of pesticide residues [30,31].

The changes in pesticide residues in the wheat and flour storage process are complex, and it is susceptible to the influence of storage conditions such as temperature and relative humidity. Therefore, the degradation pattern of pesticide residues during wheat and flour storage is mainly explored in the following study to construct the degradation model of pesticide residues in the wheat flour supply chain.

### 3.3. Study on the Degradation Pattern of Five Pesticide Residues in Wheat and Flour

The second-order mathematical models of the degradation of five pesticides in wheat and flour at different times, temperatures, and relative humidity were constructed using Minitab 17 software, and the degradation models of five pesticides at different storage conditions are shown in Table 6 and Table 7.

As can be seen in Table 6 and Table 7, the R^2^ of five pesticides is above 0.796 by building models, which indicates that the fitting result is good and reaches the expected level. By constructing the mathematical model, the degradation pattern of pesticide residues in wheat and flour under different storage conditions can be reasonably predicted.

### 3.4. Effect of Storage Conditions on the Degradation of Five Pesticides in Wheat and Flour

#### 3.4.1. Effect of Storage Temperature on the Degradation of Pesticide Residues

**Figure 4:**
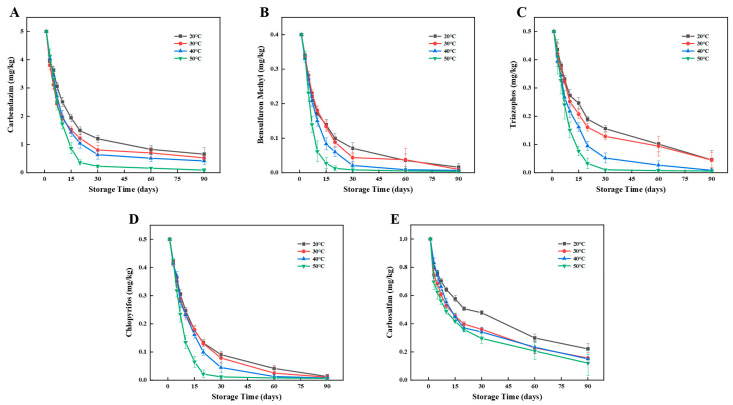
Effects of temperature on degradation of five pesticides during storage of wheat. Left to right: (**A**) Carbendazim, (**B**) Bensulfuron methyl, (**C**) Triazophos, (**D**) Chlorpyrifos, and (**E**) Carbosulfan.

**Figure 5:**
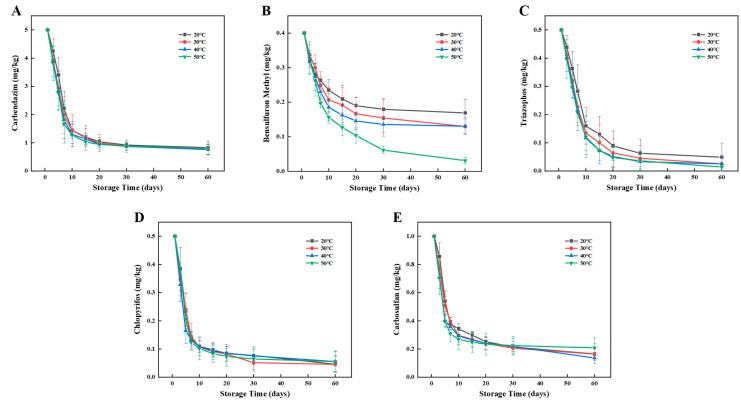
Effects of temperature on the degradation of five pesticides during storage of flour. Left to right: (**A**) Carbendazim, (**B**) Bensulfuron methyl, (**C**) Triazophos, (**D**) Chlorpyrifos, and (**E**) Carbosulfan.

The degradation patterns of pesticide residues of carbendazim, bensulfuron methyl, triazophos, chlorpyrifos, and carbosulfan in wheat and flour at different storage temperatures are shown in Figure 4 and Figure 5.

As shown in Figure 4, the five pesticide residues decreased with storage time during the 90-day storage period of wheat. Under the four temperatures from low to high, the carbendazim residue decreased by 86.83%, 89.47%, 91.63%, and 98.14% with the half-lives of 10.27, 7.33, 8.04, and 6.97 days, respectively, the bensulfuron methyl residue decreased by 96.00%, 97.63%, 98.31%, and 99.00% with the half-lives of 8.93, 9.11, 7.50, and 5.37 days respectively, the triazophos residue decreased by 90.87%, 91.05%, 98.30%, and 98.80% with the half-lives of 13.47, 11.16, 8.48, and 6.61 days, respectively, the chlorpyrifos residue decreased by 97.40%, 97.85%, 98.45%, 98.70% with the half-lives of 10.09, 9.64, 9.11, and 6.43 days, respectively, the carbosulfan residue decreased by 77.85%, 84.33%, 84.93%, and 87.95% with half-lives of 20.42, 11.87, 12.67, and 9.73 days, respectively. In conclusion, the degradation rates of the five pesticides increased with increasing temperature and reached a peak at 50 °C.

As shown in Figure 5, the five pesticide residues decreased with storage time during the 60-day storage period of flour. Under the four temperatures from low to high, the carbendazim residue decreased by 83.41%, 83.83%, 84.74%, and 84.00% with the half-lives of 6.73, 5.90, 5.55, and 5.25 days, respectively, the bensulfuron methyl residue decreased by 57.74%, 67.54%, 67.36%, and 92.25% with the half-lives of 15.23, 12.04, 9.15, and 7.91 days, respectively, the triazophos residue decreased by 90.22%, 94.92%, 94.94%, and 95.21% with the half-lives of 7.85, 6.49, 6.02, and 5.84 days, respectively, the chlorpyrifos residue decreased by 82.40%, 83.81%, 90.87%, and 98.62% with the half-lives of 4.66, 4.60, 3.78, and 4.37 days, respectively, the carbosulfan residue decreased by 83.48%, 83.58%, 86.38%, and 79.18% with half-lives of 6.08, 5.49, 4.54, and 4.25 days, respectively. In conclusion, the degradation rates of the five pesticides increased with increasing temperature and reached a peak at 50 °C.

In general, with the increase of storage temperature, the chemical reaction rate of pesticide degradation process is accelerated, and the volatility of the pesticide is enhanced, so the half-lives of all pesticide residues tested decreased. However, the susceptibility to temperature of the five pesticides are different, with carbosulfan as the most unsusceptible one. Temperature influences the volatility of pesticides, and those with lower boiling point may be more susceptible. Temperature also promotes the chemical reaction rate involved in the process of pesticide degradation, and Q_10_ coefficient of these reactions to temperature are different, and this may also explain the different susceptibility to temperature of pesticides [32].

#### 3.4.2. Effect of Relative Humidity during Storage on the Degradation of Pesticide Residues

**Figure 6:**
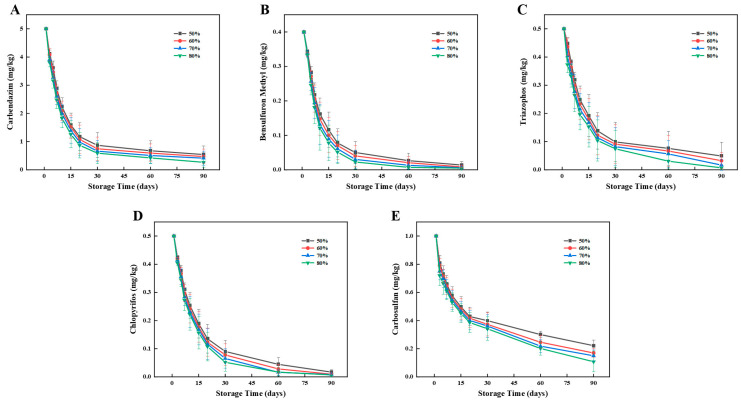
Effects of relative humidity on degradation of five pesticides during storage of wheat. Left to right: (**A**) Carbendazim, (**B**) Bensulfuron methyl, (**C**) Triazophos, (**D**) Chlorpyrifos, and (**E**) Carbosulfan.

**Figure 7:**
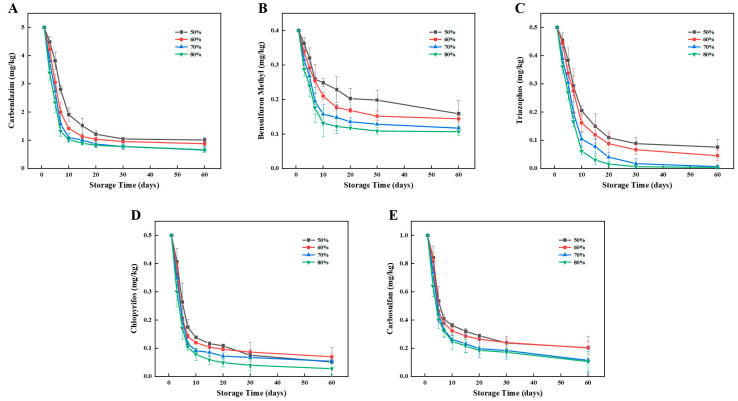
Effects of relative humidity on degradation of five pesticides during storage of flour. Left to right: (**A**) Carbendazim, (**B**) Bensulfuron methyl, (**C**) Triazophos, (**D**) Chlorpyrifos, and (**E**) Carbosulfan.

The degradation patterns of pesticide residues of carbendazim, bensulfuron methyl, triazophos, chlorpyrifos, and carbosulfan in wheat and flour at different storage relative humidity are shown in Figure 6 and Figure 7.

As shown in Figure 6, the five pesticide residues decreased with storage time during the 90-day storage period of wheat. Under the four relative humidity from low to high, the carbendazim residue decreased by 89.14%, 90.57%, 91.72%, and 94.64% with the half-lives of 8.93, 8.31, 7.68, and 7.15 days, respectively, the bensulfuron methyl residue decreased by 96.50%, 97.88%, 98.44%, and 98.75% with the half-lives of 8.39, 7.68, 7.06, and 6.61 days, respectively, the triazophos residue decreased by 90.20%, 93.50%, 96.65%, and 98.60% with the half-lives of 10.62, 9.64, 8.75, and 8.04 days, respectively, the chlorpyrifos residue decreased by 96.60%, 98.07%, 98.33%, and 98.60% with the half-lives of 10.62, 9.91, 9.29, and 8.66 days, respectively, the carbosulfan residue decreased by 77.83%, 83.00%, 85.00%, and 89.23% with half-lives of 14.01, 13.70, 12.76, and 12.05 days, respectively. In conclusion, the degradation rates of the five pesticides increased with increasing relative humidity and reached a peak at 80%.

As shown in Figure 7, the five pesticide residues decreased with storage time during the 60-day storage period of flour. Under the four relative humidity states from low to high, the carbendazim residue decreased by 79.84%, 82.46%, 86.96%, and 86.72% with the half-lives of 19.43, 11.75, 7.56, and 6.02 days, respectively, the bensulfuron methyl residue decreased by 60.32%, 63.98%, 70.75%, and 73.35% with the half-lives of 8.91, 7.56, 5.78, and 4.84 days respectively, the triazophos residue decreased by 84.91%, 90.94%, 98.70%, and 99.24% with the half-lives of 5.55, 4.54, 4.07, and 3.60 days, respectively, the chlorpyrifos residue decreased by 79.62%, 79.79%, 88.59%, and 89.42% with the half-lives of 6.20, 5.40, 4.78, and 4.19 days, respectively, the carbosulfan residue decreased by 90.79%, 90.89%, 88.88%, and 88.92% with half-lives of 6.08, 5.49, 4.54, and 4.25 days, respectively. In conclusion, the degradation rates of the five pesticides increased with increasing relative humidity and reached a peak at 80%.

In general, with the increase of storage relative humidity, the chemical reaction rate of the pesticide degradation process is accelerated, and the half-lives of all pesticide residues tested decreased. However, different structures of pesticides have different sensitivity to relative humidity. Relative humidity influences the hydrolysis reaction of pesticides, and those with more ester bonds may be more susceptible [33]. Relative humidity also promotes the growth and reproduction rate of microorganisms involved in the process of pesticide degradation, and microbial activity has an effect on the degradation process of pesticides, and this could also explain the different susceptibility to relative humidity of pesticides [34].

#### 3.4.3. Interaction between Storage Temperature and Relative Humidity on Pesticide Residue Degradation

**Figure 8:**
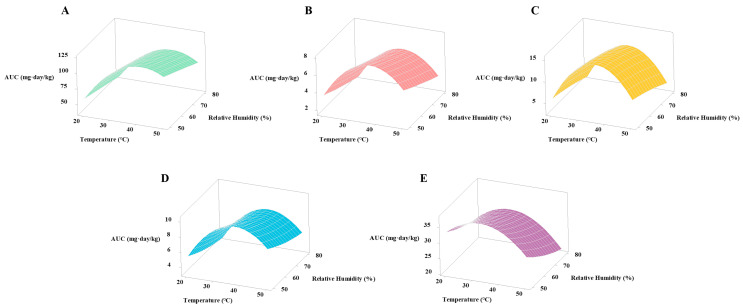
Effects of temperature and relative humidity on degradation of five pesticides during storage of wheat. Left to right: (**A**) Carbendazim, (**B**) Bensulfuron methyl, (**C**) Triazophos, (**D**) Chlorpyrifos, and (**E**) Carbosulfan.

**Figure 9:**
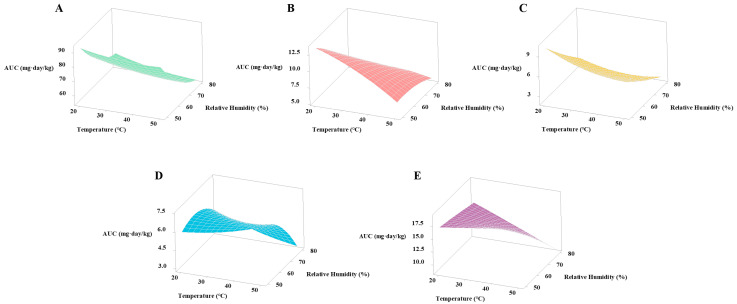
Effects of temperature and relative humidity on degradation of five pesticides during storage of flour. Left to right: (**A**) Carbendazim, (**B**) Bensulfuron methyl, (**C**) Triazophos, (**D**) Chlorpyrifos, and (**E**) Carbosulfan.

The interaction between temperature and relative humidity on degradation of pesticide residues was studied by response surface analysis using Minitab 17 software, as shown in Figure 8 and Figure 9. AUC is the area integral under the pesticide residue degradation curve for a specific combination of temperature and relative humidity, which reflects a comprehensive measure of the level of pesticide degradation with time at a specific temperature and relative humidity. The increase of AUC indicates the decrease of pesticide degradation rate, and the decrease of AUC indicates the increase of pesticide degradation rate. The interaction relationship between temperature and relative humidity on AUC of wheat stored for 90 days is shown in Figure 8, with four temperatures at 20 °C, 30 °C, 40 °C, 50 °C, and four relative humidity at 50%, 60%, 70%, 80%, respectively. The analysis showed that both temperature and relative humidity significantly (*p* < 0.05) affected the AUC of the five pesticides. With the increases of temperature and relative humidity, the AUC of five pesticides increased to a certain extent and then decreased.

The interactional relationship between temperature and relative humidity on AUC of flour stored for 60 days is shown in Figure 9, with four temperatures at 20 °C, 30 °C, 40 °C, and 50 °C, and four relative humidity at 50%, 60%, 70%, and 80%, respectively. The results showed that temperature and relative humidity could significantly (*p* < 0.05) affect the AUC of carbendazim, bensulfuron methyl, and triazophos, and relative humidity could significantly (*p* < 0.05) affect the AUC of chlorpyrifos and carbosulfan. There was a significant (*p* < 0.05) interaction between temperature and relative humidity on the AUC of triazophos, chlorpyrifos, and carbosulfan, with *p* values of 0.031, 0.032, and 0.038, respectively. The AUC of five pesticides decreased with increasing temperature and relative humidity.

The reason may be that different structure of pesticides have different sensitivity to temperature and relative humidity. In general, high temperatures make the molecular structure of pesticides vulnerable to damage, and the degradation rate of the pesticide increases with the temperature. Changes of relative humidity can affect the hydrolysis mechanism of pesticides due to interaction between lipid molecules in pesticides and water molecules, destroying the structure of pesticide molecules and leading to the degradation of pesticide residues. Therefore, pesticides with different structures have different rates of residue degradation [35].

## 4. Conclusions

In this study, the five pesticides in wheat and flour were extracted and purified by the QuEChERS method and quantified by UPLC-MS/MS. The linear range, linear equation, LOD, LOQ, recovery rate, and precision of the method were investigated, and the results showed that the method was simple, rapid, with high accuracy and good applicability.

A quantitative model was constructed to predict the pesticide residue degradation during the storage of wheat and flour. The results showed that the R^2^ reached above 0.817 in wheat, and the R^2^ reached above 0.796 in flour, with good fitting effect. The model could be used to predict the degradation of pesticide residues at given time points of the wheat flour supply chain from wheat grain to the final product, i.e., wheat flour.

In flour and wheat, the five pesticide residues gradually decreased with the increase of storage time, and the degradation rate was faster in the early stage and slower in the later stage. The degradation rates of the five pesticides increased with increasing temperature and reached a peak at 50 °C. The degradation rates of the five pesticides increased with increasing relative humidity and reached a peak at 80%. Temperature may influence the volatility of pesticides and the rate of chemical reactions involved in the degradation process. Relative humidity may influence the hydrolysis reaction of pesticides and the growth and reproduction rate of microorganisms. Results showed that high temperature and high relative humidity accelerate the degradation of the five pesticides residues, and their degradation profiles and half-lives over temperature and relative humidity varied among pesticides.

## Figures and Tables

**Table 1:** Mass spectrometric parameters of five pesticides.

Pesticide Name	Retention Time/min	Qualitative Ion Pair	Quantitative Ion Pair	Tapered Hole Voltage (V)	Collision Energy (eV)
Carbendazim	3.38	192.1–132.1	192.1–160.1	18	35
Bensulfuron Methyl	6.65	411.2–91.14	411.2–182.1	27	20
Triazophos	7.19	314.1–118.9	314.1–161.9	18	35
Chlorpyrifos	8.58	349.9–97	349.9–198	18	35
Carbosulfan	9.36	381.2–118.2	381.2–160.1	18	35

**Table 2:** Standard working curves of five pesticides in wheat blank matrix solution.

Pesticides	Linear Range (μg/L)	Linear Equation	R^2^
Carbendazim	5–2000	y = 0.1165x + 1.2126	0.9993
Bensulfuron Methyl	5–2000	y = 0.0460x − 0.0169	0.9999
Triazophos	5–2000	y = 0.3872x − 0.1041	0.9996
Chlorpyrifos	5–2000	y = 0.2607x − 0.2837	0.9995
Carbosulfan	20–2000	y = 0.0780x − 0.1619	0.9990

**Table 3:** Standard working curves of five pesticides in flour blank matrix solution.

Pesticides	Linear Range (μg/L)	Linear Equation	R^2^
Carbendazim	5−1000	y = 0.1368x + 0.0838	0.9994
Bensulfuron Methyl	5−1000	y = 0.0347x + 0.0123	0.9995
Triazophos	5−1000	y = 0.1565x + 0.2937	0.9995
Chlorpyrifos	5−1000	y = 0.0634x + 0.0762	0.9993
Carbosulfan	5−1000	y = 0.0352x + 0.0020	0.9996

**Table 4:** The matrix spike recovery, relative standard deviation, limits of detection and limits of quantification of five pesticides.

Pesticides	Recovery (%)	Precision (%)	LOD (mg/kg)	LOQ (mg/kg)
	0.05 mg/kg	0.05 mg/kg		
Carbendazim	106.28%	3.36%	0.005	0.01
Bensulfuron Methyl	92.77%	5.32%	0.002	0.005
Triazophos	96.82%	6.62%	0.001	0.003
Chlorpyrifos	97.69%	2.88%	0.001	0.003
Carbosulfan	86.77%	6.76%	0.001	0.002

**Table 5:** Critical points in wheat flour supply chain.

Critical Points	Main Mechanisms	IMPACT FACTORS
Raw material storage	Pesticide residue degradation	Storage time (t_w_), Temperature (T_w_), Relative humidity (RH_w_)
Milling	Physical removal of the cortex and Pesticide residue degradation	Mass fraction of pesticide residues in the cortex
Final product storage	Pesticide residue degradation	Storage time (t_f_), Temperature (T_f_), Relative humidity (RH_f_)

**Table 6:** A mathematical model for predicting the change of five pesticides during wheat storage.

Pesticide Name	Prediction Model	R^2^
Carbendazim	C_1_/C_0_ = 0.438 − 0.00246t_w_ + 0.0217T_w_ − 0.00184RH_w_ + 0.0000736t_w_^2^ − 0.000312T_w_^2^ − 0.00004015(RH_w_)^2^ + 0.0000674T_w_t_w_ + 0.00000585T_w_RH_w_ + 0.00005695T_w_RH_w_	0.830
Bensulfuron Methyl	C_1_/C_0_ = 0.5 − 0.034575t_w_ + 0.035875T_w_ − 0.007RH_w_ + 0.000285t_w_^2^ − 0.00047T_w_^2^ + 0.000035(RH_w_)^2^ + 0.000025T_w_t_w_ − 0.00003t_w_RH_w_ + 0.000001T_w_RH_w_	0.817
Triazophos	C_1_/C_0_ = 0.298 − 0.02986t_w_ + 0.046T_w_ − 0.00346RH_w_ + 0.000244t_w_^2^ − 0.000666T_w_^2^ − 0.000004(RH_w_)^2^ − 0.000008t_w_T_w_ + 0.000002t_w_RH_w_ + 0.000036T_w_RH_w_	0.852
Chlorpyrifos	C_1_/C_0_ = 0.586 − 0.03286t_w_ + 0.02572T_w_ − 0.00434RH_w_ + 0.000276t_w_^2^ − 0.000328T_w_^2^ + 0.000016(RH_w_)^2^ − 0.000028t_w_T_w_ + 0.00001t_w_RH_w_ + 0.000006T_w_RH_w_	0.868
Carbosulfan	C_1_/C_0_ = 0.728 − 0.02008t_w_ + 0.01628T_w_ − 0.00171RH_w_ + 0.000157t_w_^2^ − 0.000259T_w_^2^ − 0.000007(RH_w_)^2^ + 0.000019t_w_T_w_ − 0.000025t_w_RH_w_ − 0.000017T_w_RH_w_	0.863

Note: T_w_, wheat storage temperature; t_w_, wheat storage time; RH_w_, wheat storage relative humidity; C_0_, wheat initial pesticide residue concentration; C_1_, wheat pesticide residue concentration after storage.

**Table 7:** A mathematical model for predicting the change of five pesticides during flour storage.

Pesticide Name	Prediction Model	R^2^
Carbendazim	C_3_/C_2_ = 1.756 − 0.05176t_f_ − 0.0042T_f_ − 0.01902RH_f_ + 0.000575t_f_^2^ + 0.000043T_f_^2^ + 0.0001(RH_f_)^2^ + 0.0000412t_f_T_f_ + 0.0000768t_f_RH_f_ − 0.0000162T_f_RH_f_	0.796
Bensulfuron Methyl	C_3_/C_2_ = 1.738 − 0.05116t_f_ + 0.0033T_f_ + 0.0185RH_f_ + 0.000549t_f_^2^ − 0.000095T_f_^2^ + 0.00007(RH_f_)^2^ + 0.000006T_f_t_f_ + 0.000116t_f_RH_f_ + 0.000081T_f_RH_f_	0.802
Triazophos	C_3_/C_2_ = 1.253 − 0.0411t_f_ − 0.0022T_f_ − 0.006RH_f_ + 0.000491t_f_^2^ + 0.00021T_f_^2^ − 0.0000165(RH_f_)^2^ + 0.0000065t_f_T_f_ + 0.0000115t_f_RH_f_ + 0.0001T_f_RH_f_	0.878
Chlorpyrifos	C_3_/C_2_ = 0.968 − 0.05488t_f_ − 0.0106T_f_ − 0.00196RH_f_ + 0.00063t_f_^2^ − 0.000192T_f_^2^ + 0.00002(RH_f_)^2^ + 0.000018t_f_T_f_ + 0.0000656t_f_RH_f_ + 0.000005T_f_RH_f_	0.840
Carbosulfan	C_3_/C_2_ = 1.314 − 0.03604t_f_ + 0.01218T_f_ − 0.01RH_f_ + 0.000467t_f_^2^ + 0.000225T_f_^2^ + 0.0000344(RH_f_)^2^ + 0.000023t_f_T_f_ − 0.000045t_f_RH_f_ − 0.000008T_f_RH_f_	0.863

Note: T_f_, flour storage temperature; t_f_, flour storage time; RH_f_, flour storage relative humidity; C_2_, flour initial pesticide residue concentration; C_3_, flour pesticide residue concentration after storage.

## Data Availability

The data presented in this study are available on request from the corresponding author.
